# Carbapenem-hydrolyzing Oxacillinase-48 and Oxacillinase-181 in Canada, 2011

**DOI:** 10.3201/eid1901.120706

**Published:** 2013-01

**Authors:** Laura F. Mataseje, David A. Boyd, Linda Hoang, Miguel Imperial, Brigitte Lefebvre, Mark Miller, Susan M. Poutanen, Diane Roscoe, Barbara M. Willey, Michael R. Mulvey

**Affiliations:** Author affiliations: National Microbiology Laboratory, Winnipeg, Manitoba, Canada (L.F. Mataseje, D.A. Boyd, M.R. Mulvey);; British Columbia Center for Disease Control, Vancouver, British Columbia, Canada (L. Hoang, M. Imperial);; Laboratoire de Santé Publique du Québec, Sainte-Anne-de-Bellevue, Quebec, Canada (B. Lefebvre);; SMBD–Jewish General Hospital, Montreal, Quebec, Canada (M. Miller);; University of Toronto, Toronto, Ontario, Canada (S.M. Poutanen);; University Health Network/Mount Sinai Hospital, Toronto (S.M. Poutanen, B.M. Willey);; Vancouver General Hospital, Vancouver (D. Roscoe)

**Keywords:** carbapenemases, oxacillinase-48, oxacillinase-181, OXA-48, OXA-181, hydrolyzing, Canada, Enterobacteriaceae, Klebsiella pneumoniae, antimicrobial resistance, bacteria

**To the Edito**r**:** In 2001, a *Klebsiella pneumoniae* isolate from a patient in Turkey was found to harbor a novel class D carbapenem-hydrolyzing oxacillinase, OXA-48 (*1*). Although this enzyme hydrolyzes carbapenems at a low level and shows weak activity against expanded-spectrum cephalosporins, it is often associated with other β-lactamases and is multidrug resistant. Reports of *Enterobacteriaceae* harboring OXA-48 have been described across Europe, the Mediterranean area, and the Middle East (*2*). In addition, OXA-181, which differs from OXA-48 by 4 aa substitutions, has been described in India (*2*). We describe the emergence of OXA-48 and OXA-181 in Canada.

Hospital and provincial public health laboratories in Canada voluntarily submitted *Enterobacteriaceae* isolates to the National Microbiology Laboratory. Isolates submitted by the laboratories were not susceptible to carbapenems and were to be tested by PCR for carbapenemase genes (KPC, NDM, IMP, VIM, OXA-48, and GES) (*3*). During April–November 2011, a total of 4 isolates (3 *K. pneumoniae*, 1 *Escherichia coli*) tested positive for the *bla*_OXA-48_–type gene. Sequencing, using the primers preOXA-48A and -48B (*4*), revealed that 3 of the isolates (*K. pneumoniae* 11-882 and 11-2720 and *E. coli* 11-1498) possessed the *bla*_OXA-48_ gene, and the other isolate (*K. pneumoniae* 11-2568) possessed the *bla*_OXA-181_ gene. We conducted additional β-lactamase PCR and sequencing as described (*3*) (Table). The Modified Hodge test (using a 10-μg disk of ertapenem and meropenem) showed that all isolates were strongly positive for carbapenemase production.

**Table:** Patient data and antimicrobial drug susceptibility profiles for isolates of *Klebsiella pneumoniae* and *Escherichia coli* harboring OXA-type carbapenemases, Canada, 2011*

Variable	Bacterial isolate, strain no., OXA-type								*E. coli* DH10B†
	*K. pneumoniae*				*E. coli *		Plasmid		
	11-882, OXA-48	11-2568, OXA-181	11-2720, OXA-48		11-1498, OXA-48		p48-11-882	p181-11-2568	
Patient data							NA	NA	NA
Recent travel	NK	India	Lebanon		Dubai		NA	NA	NA
Site of bacterial isolation	Urine	Perirectal	Urine		Skin swab		NA	NA	NA
Infection/colonization	NK	Colonization	Infection		Colonization		NA	NA	NA
Hospitalization	NK	General ward	Outpatient		ICU		NA	NA	NA
Bacterial isolate data									
β-lactamase	TEM-1, SHV-11	TEM-1, SHV-11, CTX-M-15, OXA-1	SHV-11, OXA-1		TEM-1, CTX-M-24		TEM-1	Neg	NA
Sequence type	395	147	831		38		NA	NA	NA
Antimicrobial drug susceptibility test, drug‡									
Vitek2,§ MIC μg/mL									
Amikacin	<2.0	4.0	<2.0		<2.0		<2.0	<2.0	<2.0
Aztreonam	<1.0	>64.0	<1.0		32.0		<1.0	<1.0	<1.0
Cefazolin	32.0	>64.0	16.0		>64.0		>64.0	32.0	<4.0
Cefepime	<1.0	>64.0	<1.0		<1.0		<1.0	<1.0	<1.0
Ceftriaxone	<1.0	>64.0	<1.0		>64.0		<1.0	<1.0	<1.0
Ciprofloxacin	>4.0	>4.0	<0.25		<0.25		<0.25	<0.25	<0.25
Ertapenem	>8.0	>8.0	4.0		4.0		>8.0	4.0	<0.5
Gentamicin	<1.0	>16.0	<1.0		>16.0		<1.0	<1.0	<1.0
Imipenem	4.0	>16.0	<1.0		<1.0		8.0	2.0	<1.0
Meropenem	1.0	>16.0	<0.25		0.5		8.0	<0.25	<0.25
Tigecycline¶	1.0	2.0	<0.5		<0.5		<0.5	<0.5	<0.5
TMP/SXT	>320.0	>320.0	<20.0		>320.0		>320	<20	<20.0
Etest,§ MIC μg/mL									
Imipenem	1.0	24.0	0.38		0.38		0.5	0.5	0.19
Meropenem	0.5	24.0	0.19		0.38		0.125	0.19	0.032
Ertapenem	2.0	>32.0	0.5		2.0		0.38	0.75	0.008
Colistin#	0.38	0.5	0.5		0.5		0.125	0.125	ND
Disk diffusion,** zone diameter, mm									
Imipenem	20	8	23		22		24	25	33
Meropenem	20	7	23		22		22	28	35
Ertapenem	16	6	19		16		23	22	35

*NK, not known; NA, not applicable; ICU, intensive care unit; Neg, negative; TMP/SXT, trimethoprim/sulfamethoxazole; ND, not done. †OXA-48–type plasmids were electroporated into *E. coli* DH10B cells. ‡Additional antimicrobial drugs on panel: ampicillin, ampicillin/sulbactam, nitrofurantoin, piperacillin/tazobactam, tobramycin.  §Test from bioMérieux Canada Inc., St. Laurent, Quebec, Canada. ¶MIC breakpoints for tigecycline were based on the US Food and Drug Administration criteria for *Enterobacteriaceae*: susceptible, <2 μg/mL; intermediate, 4 μg/mL; resistant, >8 μg/mL. #MIC breakpoints for colistin followed Clinical Laboratory Standards Institute breakpoint criteria for *Acinetobacter* spp.: susceptible, MIC <2 μg/mL; resistant, MIC >4 μg/mL. **Test from Clinical Laboratory Standards Institute (www.clsi.org/source/orders/free/m100-s22.pdf).

The Table shows clinical data and antimicrobial drug susceptibility profiles for the *K. pneumoniae* and *E. coli* harboring OXA-type carbapenemases. Half of the isolates were from men >65 years of age. Three of the case-patients had a history of travel to regions where OXA-48–type carbapenemases are endemic (Lebanon, India, and Dubai), and while still abroad (shortly before being hospitalized in Canada), 2 case-patients had sought medical attention.

Pulsed-field gel electrophoresis of 3 *K. pneumoniae* isolates digested with *Xba*I showed unique fingerprint patterns (data not shown). Multilocus sequence typing (www.pasteur.fr/recherche/genopole/PF8/mlst/Kpneumoniae.html) of *K. pneumoniae* 11-2568, 11-882, and 11-2720 showed that they belonged to sequence type (ST) 147, ST395, and ST831, respectively. ST395 has been identified in *K. pneumoniae* from Morocco, Amsterdam, and France harboring *bla*_OXA-48_ (*2*). *E. coli* 11-1498 was shown to belong to sequence type 38, previously described in a nosocomial outbreak and in isolated cases of OXA-48 in France; travel to Morocco and Egypt was linked to those cases (*5*,*6*). Phylogenetic grouping of the *E. coli* isolate revealed that it belonged to group D, which is associated with virulent extraintestinal strains (*7*).

Antimicrobial drug susceptibilities were determined by using Vitek 2 (GN-25) (bioMérieux, Canada Inc., St Laurent, Quebec, Canada) and interpreted by using the 2012 guidelines (M100-S22) of the Clinical Laboratory Standards Institute (www.clsi.org/source/orders/free/m100-s22.pdf) (Table). All but 1 isolate (*K. pneumoniae* 11-2720) was multidrug resistant (defined as resistant to >3 antimicrobial drug classes). All isolates were resistant to ampicillin, cefazolin, and pipericillin/tazobactam. Two isolates were resistant to broad-range cephalosporins, both of which contained the CTX-M–type extended-spectrum β-lactamase. All isolates were also resistant to ertapenem, 2 of 4 were resistant to imipenem, and 1 was resistant to meropenem. Results were confirmed by Etest (bioMérieux, Canada Inc.) and Clinical Laboratory Standards Institute disk diffusion, with the exception of results for *K. pneumoniae* 11-882, which showed susceptibility to meropenem and imipenem by Etest and intermediate susceptibility by disk diffusion (Table). All isolates were susceptible to tigecycline and colistin.

PCR mapping, using previously described primers (*8*), identified the *bla*_OXA-48_ gene located on the transposon Tn*1999*. The *bla*_OXA-181_ gene was found downstream of ISE*cp1*, as described (*4*). We isolated plasmid DNA by using QIAGEN Plasmid Mini Kits (QIAGEN, Mississauga, ON, Canada) and attempted to transfer the plasmid harboring the *bla*_OXA-48_–type gene, using electroporation into *E. coli* DH10B, as described (*3*). Plasmid transfer of *bla*_OXA-48_–type genes was successful only for *K. pneumoniae* 11-882 and 11-2568. PCR revealed the transfer of *bla*_TEM-1_ and *bla*_OXA-48_ along with a plasmid of ≈114 Kb (p48-11-882). In addition, *bla*_OXA-181_ was transferred along with a plasmid of ≈4 Kb (p181-11-2568) with no additional β-lactamases present.

Replicon typing (*9*,*10*), using primers for incompatibility group IncR (IncRrv 5′-GTGTGCTGTGGTTATGCCTCA-3′), showed that p48-11-882 belonged to IncR and to the OXA-48–associated IncL/M. Plasmid p181-11-2568 was negative for all replicons tested.

Transformants were resistant to ampicillin, cefazolin, and pipericillin/tazobactam but susceptible to third- and fourth-generation cephalosporins. This finding is not surprising because OXA-48 and OXA-181 show weak activity against expanded-spectrum cephalosporins (*1*,*4*). Resistance to third- and fourth-generation cephalosporins in clinical isolates is likely caused by a combination of additional mechanisms, such as porin mutations, or additional β-lactamases, such as CTX-M–type, extended-spectrum β-lactamases (Table).

We report the emergence of OXA-48 and OXA-181 in North America. The emergence of carbapenem-resistant *Enterobacteriaceae* in Canada is of concern because they are difficult to detect in the laboratory, and treatment options are lacking. The history of travel by the patients in our study to regions where carbapenem-resistant *Enterobacteriaceae* are endemic highlights the necessity for understanding the potential risk factors associated with acquiring these multidrug-resistant pathogens.
